# Safety and efficacy of short-term dual antiplatelet therapy combined with intensive rosuvastatin in acute ischemic stroke

**DOI:** 10.1016/j.clinsp.2023.100171

**Published:** 2023-02-03

**Authors:** Ting Deng, Wei He, Xiaohua Yao, Jingmian Chen, Xiaomeng Liu, Lushan Liu, Tong Zhang, Haitao Lu

**Affiliations:** a Emergency Department, China Rehabilitation Research Center Beijing Bo'ai Hospital, Beijing, China; b Neurology Department, China Rehabilitation Research Center Beijing Bo'ai Hospital, Beijing, China

**Keywords:** Short-Term Dual Antiplatelet Therapy, Single Antiplatelet Therapy, Intensive Rosuvastatin, Recurrent Ischemic Stroke, AIS, Acute Ischemic Stroke, DAPT, Dual Antiplatelet Therapy, TIA, Transient Ischemic Attack, SAPT, Single Antiplatelet Therapy, CT, Computed Tomography, MRI, Magnetic Resonance Imaging, NIHSS, National Institutes of Health Stroke Scale, bNIHSS, the baseline scores of National Institutes of Health Stroke Scale, OMT, Time of Onset to Medication, GUSTO, Global Use of Streptokinase and Tissue plasminogen activator to treat coronary Occlusion, SILI, Statin-Induced Liver Injury, SAM, Statin-Associated Myopathy, ALT, Alanine Aminotransferase, AST, Aspartate Aminotransferase, CK, Creatine Kinase, LDH, Lactate Dehydrogenase, IQR, Interquartile Range, SBP, Systolic Blood Pressure, DBP, Diastolic Blood Pressure, ASCVD, Atherosclerotic Cardiovascular Disease, eNOS, Endothelial Nitric Oxide Synthase

## Abstract

**Objective:**

To investigate the safety and efficacy of short-term (7-day) Dual Antiplatelet Therapy (DAPT) with intensive rosuvastatin in Acute Ischemic Stroke (AIS).

**Methods:**

In this study, patients with AIS in the emergency department of the hospital from October 2016 to December 2019 were registered and divided into the control group (Single Antiplatelet Therapy [SAPT] + rosuvastatin) and the study group (7-day DAPT + intensive rosuvastatin) according to the therapy regimens. The generalized linear model was used to compare the National Institute of Health Stroke Scale (NIHSS) scores between the two groups during the 21-day treatment. A Cox regression model was used to compare recurrent ischemic stroke, bleeding events, Statin-Induced Liver Injury (SILI), and Statin-Associated Myopathy (SAM) between the two groups during the 90-day follow-up.

**Results:**

Comparison of NIHSS scores after 21-day treatment: NIHSS scores in the study group decreased significantly, 0.273-times as much as that in the control group (Odds Ratio [OR] 0.273; 95% Confidence Interval [95% CI] 0.208–0.359; p < 0.001). Comparison of recurrent ischemic stroke during the 90-day follow-up: The therapy of the study group reduced the risk of recurrent stroke by 65% (7.76% vs. 22.82%, Hazard Ratio [HR] 0.350; 95% CI 0.167–0.730; p = 0.005). Comparison of bleeding events: There was no statistical difference between the two groups (7.79% vs. 6.71%, HR = 1.076; 95% CI 0.424–2.732; p = 0.878). No cases of SILI and SAM were found.

**Conclusions:**

Short-term DAPT with intensive rosuvastatin effectively relieved the clinical symptoms and significantly reduced the recurrent stroke for patients with mild-to-moderate AIS within 90 days, without increasing bleeding events, SILI and SAM.

## HIGHLIGHTS

**What Is New?**

• Short-term (7-day) DAPT with intensive rosuvastatin can quickly and effectively relieve the clinical symptoms for patients with mild-to-moderate AIS within 21-day.
• Short-term (7-day) DAPT with intensive rosuvastatin significantly can reduce recurrent ischemic stroke for patients with mild-to-moderate AIS within 90-day.
• Short-term (7-day) DAPT with intensive rosuvastatin rarely increased bleeding events, statin-induced liver injury, or statin-associated myopathy in patients with mild-to-moderate AIS.

**What are the clinical implications?**

• Short-term (7-day) DAPT with intensive rosuvastatin might be a good alternative therapy regimen for mild-to-moderate AIS.

## Introduction

Stroke is a major chronic non-communicable disease that is a serious health threat among Chinese people and is the leading cause of death and disability in adults in China.^1^ China has the highest number of stroke patients in the world.^2^ Among them, ischemic stroke accounts for 85% of all stroke, and its annual recurrence rate is as high as 5.6%,^3^ which has caused huge psychological pressure and economic burden to society and families.^2^ Prevention and treatment of stroke and reduction of its recurrence have become the most important topic in the field of stroke. Recently, great progress has been made in endovascular therapy, including intravenous thrombolysis and arterial thrombolysis,^4^^,^^5^ but most patients miss the optimal time window for treatment. The antiplatelet aggregation has become the most routine regimen for acute treatment and secondary prevention for Acute Ischemic Stroke (AIS).^2^

The CHANCE^6^ study initiated Dual Antiplatelet Therapy (DAPT) for AIS in China, which reduced the risk of recurrent ischemic stroke within 90 days and increased the risk of bleeding events^7^^,^^8^ in patients with minor AIS or high-risk Transient Ischemic Attack (TIA), in particular, the risk of bleeding was significantly increased after 1 week of treatment.^9^ To explore a more accurate DAPT regimen for AIS, the authors compared the safety and efficacy of short-term (7-day) DAPT+intensive rosuvastatin & Single Antiplatelet Therapy (SAPT)+rosuvastatin in patients with mild-to-moderate AIS.

## Materials and methods

### Patients

The authors registered patients with AIS admitted to the emergency department of the hospital from October 2016 to December 2019, and their diagnosis met the 2018 criteria of the Chinese Guidelines for Diagnosis and Treatment of Acute Ischemic Stroke.^10^ The study was approved by the Medical Ethics Committee of the hospital (No. 2018-022-1). The patients themselves or their legal proxies were informed of the study details, and their written consent was obtained.

Inclusion criteria: 1) Age ≥18 years; 2) Head Computed Tomography (CT) and brain Magnetic Resonance Imaging (MRI) showing new infarction lesion; 3) Definite symptoms of focal neurological deficit, and the baseline scores of National Institutes of Health Stroke Scale (bNIHSS) at registration ≤10; 4) Time of Onset to Medication (OMT) ≤ 72h.

Exclusion criteria: 1) Patients with intravenous thrombolysis/arterial thrombectomy; 2) Patients with tumors or severe functional impairment of other organs; 3) Patients on anticoagulant medication; 4) Menstruating and/or pregnant women and those planning to conceive within 3-months.

Removal Criteria: to ensure that National Institutes of Health Stroke Scale (NIHSS) scores are not affected by physical function, the authors further ruled out: 1) Age ≥ 80 years, and bNIHSS ≤ 3; 2) 70 years ≤ age < 80 years, and bNIHSS ≤ 2; 3) Age ≤ 60 years, and bNIHSS = 1; and 4) Incomplete data.

### Treatment and grouping regimens

The patients enrolled in this study were divided into a study group and a control group according to the therapy regimens. The therapy regimen for the study group was 7-day DAPT+intensive rosuvastatin, while that for the control group was SAPT + rosuvastatin. The specific regimen is as described below:

Study group: aspirin (BayerHealthCareManufacturingS.r.l.) 100 mg/d with an initial dose 300 mg for 90 days, clopidogrel (SanofiClirSNC) 75 mg/d with an initial dose 75–300 mg according to patients’ symptoms for 7 days, and rosuvastatin (Nanjing Chia Tai-Tianqing Pharmaceutical Co., Ltd.) 20 mg/d for 21 days, followed by 10 mg/d until the 90^th^ day.

Control group: aspirin 100 mg/d or clopidogrel 75 mg/d for 90 days, and rosuvastatin 10 mg/d for 90 days.

The basic treatments for the two groups were the same. Both groups were followed-up for 90 days.

### Assessment of results

Clinical efficacy: NIHSS was used to evaluate the severity of neurological deficit symptoms of AIS patients at seven-time points before treatment (T_0_), and 12h (T_1_), 24h (T_2_), 48h (T_3_), 1 week (T_4_), 2 weeks (T_5_), and 3 weeks (T_6_) after treatment. The score ranged from 0 to 43, with a higher score indicating more severe disease. Scores of ≤ 4, 5–14, and > 15, respectively, indicated mild, moderate, and severe AIS.^11^ Because there were few patients with severe AIS in the emergency department of the studied hospital, and most of them needed intravenous thrombolysis or arterial thrombectomy, only patients with bNIHSS ≤ 10 were registered in this study.

Recurrent ischemic stroke was identified in patients with AIS had another ischemic stroke within the 90-day follow-up, and brain MRI showed a new infarct or the original infarct was significantly enlarged. Regarding bleeding events, patients with AIS had bleeding events within 90 days after treatment, including intracranial hemorrhage verified by head CT, or gastrointestinal or mucocutaneous bleeding verified by a positive occult blood test. Mild, moderate, or severe bleeding was defined according to the Global Use of Streptokinase and Tissue plasminogen activator to treat coronary Occlusion (GUSTO).^12^ Statin-Induced Liver Injury (SILI) was defined when the level of Alanine Aminotransferase (ALT) or Aspartate Aminotransferase (AST) increased to ≥ 3 times higher than normal during the use of statins.^13^ Statin-Associated Myopathy (SAM) was defined when the level of Creatine Kinase (CK) rose ≥ 3 (3–10) times higher than normal during the use of statins.^14^

### Data collection

The general clinical data; medical history (hypertension, diabetes, hyperlipidemia, atrial fibrillation, prior ischemic stroke, prior antiplatelet); smoking; drinking; OMT; and NIHSS scores at seven different time points were recorded. Laboratory items included serum levels of ALT, AST, Lactate Dehydrogenase (LDH), and CK before and 2 weeks (14 ± 3 days) after treatment. Recurrent ischemic stroke events, bleeding events, and SILI and SAM events within 90 days of follow-up were recorded.

### Statistical analysis

SPSS 25.0 statistical software (IBM Corporation, Armonk, NY, USA) was used for data analysis: 1) Because the continuous data of the baseline characteristics of the two groups of patients did not conform to the normal distribution, they were expressed as median (Interquartile Range [IQR]) and were tested using the rank sum test. Categorical data were expressed as (%) and were tested using the chi-square method. 2) The generalized linear model was used to analyze the changing trend of the NIHSS scores at seven different time points. 3) A Cox proportional hazard model was used to compare the difference between recurrent ischemic stroke events and bleeding events within 90 days between the two groups; p < 0.05 was considered to indicate statistically significant differences.

## Results

### Comparison of baseline data and the levels of ALT, AST, LDH, and CK between the two groups

A total of 265 patients were enrolled in this study: 149 patients in the control group and 116 patients in the study group. There was no significant difference between the two groups in terms of age; sex; Systolic Blood Pressure (SBP) and Diastolic Blood Pressure (DBP) at registration; OMT; bNIHSS; medical history; and levels of ALT, AST, LDH, and CK before and 2 weeks (14 ± 3 days) after treatment (p > 0.05) (Table 1).

**Table 1:** Baseline characteristics of the patients^a^.

Characteristics	Control group (n = 149)	Study group (n = 116)	p-value
Median age (IQR), years	65.00 (58.50‒77.00)	65.00 (58.25‒74.50)	0.843
Female, n (%)	48 (32.2)	26 (22.4)	0.097
Median SBP (IQR), mm/Hg	154.00 (138.00‒174.00)	153.00 (142.25‒172.00)	0.887
Median DBP (IQR), mm/Hg	89.00 (78.00‒103.00)	90.00 (81.00‒103.00)	0.221
Medical history, n (%)			
Hypertension	129 (86.6)	95 (81.9)	0.309
Diabetes	64 (43.0)	46 (39.7)	0.617
Known atrial fibrillation	23 (15.4)	11(9.5)	0.195
Ischemic stroke	66 (44.3)	42 (36.2)	0.208
Antiplatelet	37 (24.8)	31 (26.7)	0.777
Median OMT (IQR) – hours	12.00 (5.00‒25.00)	18.00 (5.00‒36.00)	0.612
Median bNIHSS (IQR)	4.00 (2.00‒5.00)	4.00 (3.00‒5.00)	0.151
Median various enzymology before medication (IQR), U/L			
ALT	17.00 (12.20‒22.00)	18.35 (13.83‒24.53)	0.171
AST	16.40 (12.25‒22.10)	16.55 (13.00‒21.60)	0.751
LDH	176.00 (158.00‒200.50)	178.50 (150.50‒202.50)	0.980
CK	81.00 (55.50‒127.00)	70.00 (53.25‒109.25)	0.103
Median of various enzymology in 2 weeks after medication (IQR), U/L			
ALT	17.60 (12.05‒25.10)	17.20 (12.65‒25.58)	0.785
AST	17.00 (13.20‒21.65)	17.70 (14.10‒21.85)	0.569
LDH	181.00 (156.00‒210.00)	171.50 (151.00‒202.25)	0.056
CK	71.00 (49.00‒101.50)	68.00 (46.50‒101.50)	0.409

a There were no significant differences between the study group and control group for any parameter. SBP, Systolic Blood Pressure; DBP, Diastolic Blood Pressure; OMT, Time of Onset to Medication; bNIHSS, the baseline scores of National Institutes of Health Stroke Scale at registration; ALT, Alanine Aminotransferase; AST, Aspartate Aminotransferase; LDH, Lactate Dehydrogenase; CK, Creatine Kinase.

### Comparison of NIHSS scores between the two groups

**Figure 1:**
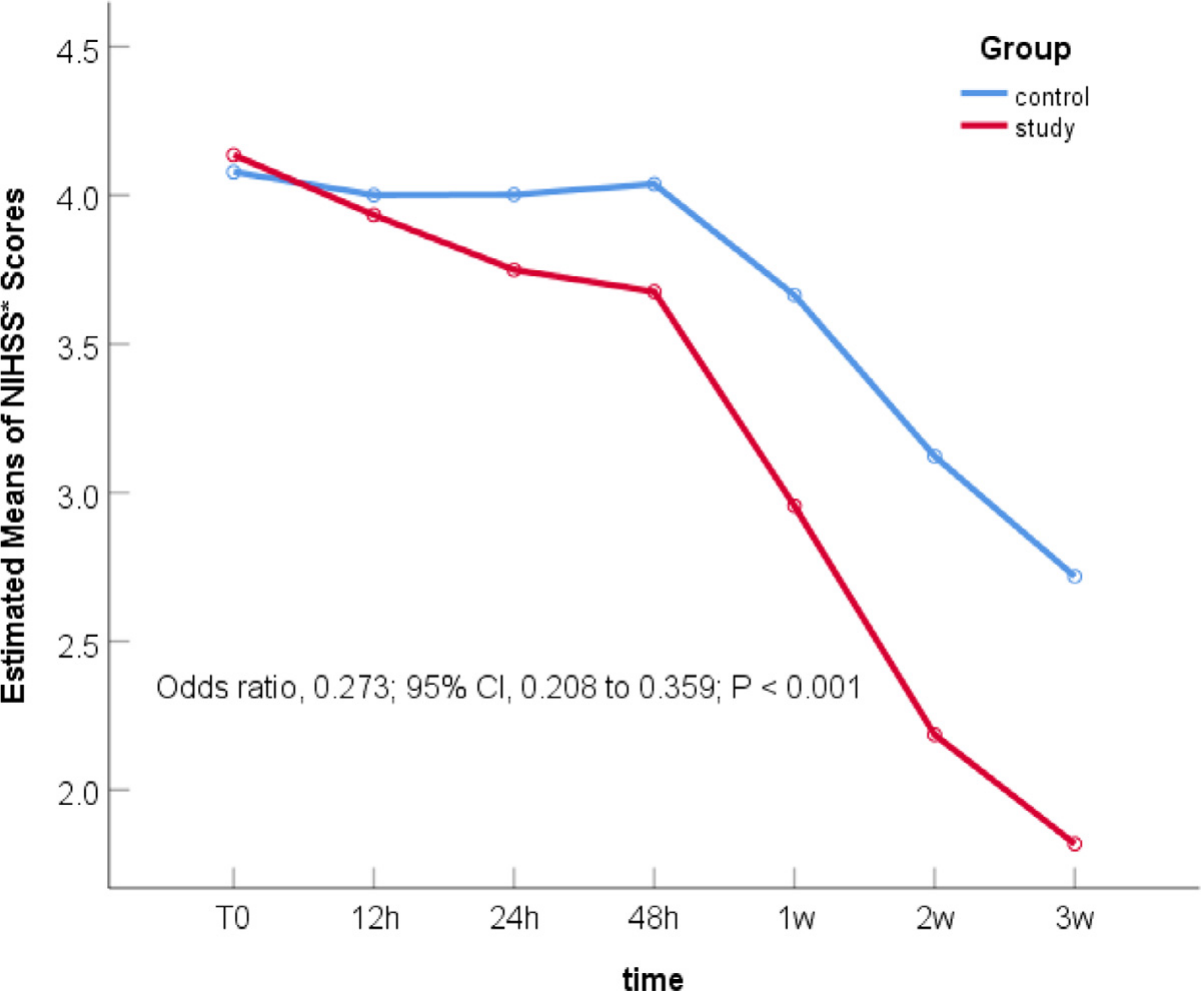
The change trend curve of NIHSS scores of the two groups within 21 days. (NIHSS, National Institutes of Health Stroke Scale at registration).

The generalized linear model was used to compare the NIHSS scores of the two groups at seven different time points before treatment (T_0_), and 12h (T_1_), 24h (T_2_), 48h (T_3_), 1 week (T_4_), 2 weeks (T_5_), and 3 weeks (T_6_) after treatment. The results are as follows:

The main effect of the therapy regimens: There was no significant difference in bNIHSS between the two groups (Odds Ratio [OR] 1.186; 95% Confidence Interval [95% CI] 0.592–2.377; p = 0.631). After 21 days of treatment, there was a statistically significant difference between the two groups, and the NIHSS score of the study group was significantly reduced, which was 27.3% of that of the control group (OR = 0.273; 95% CI 0.208–0.359; p < 0.001) (Table 2). Figure 1 was the declining trend curves of the NIHSS scores of the two groups after adjusting for the influence of age and OMT. The curves showed that the NIHSS scores of the study group were significantly lower than that of the control group after 21 days of treatment, suggesting that 7-day DAPT+intensive rosuvastatin effectively alleviated the patient's clinical symptoms.

**Table 2:** Comparison of NIHSS scores between the two groups before and 21 days after treatment.

	Coefficient	p-value	OR	95% Confidence Interval	
				Lower	Upper
(Intercept)	3.857	0.000	47.330	39.527	56.673
[study group] * [time = 0]^a^	0.171	0.631	1.186	0.592	2.377
[control group] * [time = 0]	0		1		
[study group] * [time = 6]^b^	-1.298	0.000	0.273	0.208	0.359
[control group] * [time = 6]	0		1		

a Comparison of the bNIHSS between the two groups

b Comparison of NIHSS scores between the two groups after 21 days of treatment.

Comparison of NIHSS scores within each group: With their respective bNIHSS as a reference, the NIHSS scores at the other six time points are compared to obtain their respective p-values. The NIHSS scores of the study group (Table 3) decreased significantly after 12h of treatment (OR = 0.528; 95% CI 0.295–0.948; p = 0.032), and the NIHSS score decreased more significantly after 24h of treatment (OR = 0.381; 95% CI 0.212–0.683; p = 0.001). In the control group (Table 3), only the NIHSS score after 3-weeks of treatment decreased significantly (OR = 0.404, 95% CI 0.193–0.846; p = 0.016). The curve trend chart in Figure 1 shows that compared with the control group, the NIHSS scores of the study group decreased more steeply, suggesting that 7-day DAPT+intensive rosuvastatin quickly relieved the clinical symptoms of patients.

**Table 3:** Comparison of the trend of NHISS scores in the two groups within 21 days.

	Coefficient	p-value	OR	95% Confidence Interval	
				Lower	Upper
(Intercept)	1.000	0.000	2.718	1.614	4.578
[study group] * [time = 6]	-3.103	0.000	0.045	0.025	0.081
[study group] * [time = 5]	-2.793	0.000	0.061	0.034	0.110
[study group] * [time = 4]	-2.138	0.000	0.118	0.066	0.211
[study group] * [time = 3]	-1.172	0.000	0.310	0.173	0.555
[study group] * [time = 2]	-0.966	0.001	0.381	0.212	0.683
[study group] * [time = 1]	-0.638	0.032^a^	0.528	0.295	0.948
[study group] * [time = 0]	0		1		
[control group] * [time = 6]	-0.906	0.016^b^	0.404	0.193	0.846
[control group] * [time = 5]	-0.537	0.154	0.585	0.279	1.223
[control group] * [time = 4]	0.060	0.873	1.062	0.508	2.223
[control group] * [time = 3]	0.376	0.319	1.456	0.696	3.048
[control group] * [time = 2]	0.262	0.487	1.299	0.621	2.719
[control group] * [time = 1]	0.215	0.569	1.240	0.592	2.594
[control group] * [time = 0]	0		1		

a Indicates the NIHSS score of patients after 12h of treatment (T_1_) in the study group decreased significantly, which was statistically significant compared with bNIHSS (p = 0.032).

b Indicates that the NIHSS score of the control group decreased significantly after 3 weeks of treatment (T_6_), which was statistically significant compared with bNIHSS (p = 0.016).

### Comparison of recurrent ischemic stroke between the two groups in 90 days

**Figure 2:**
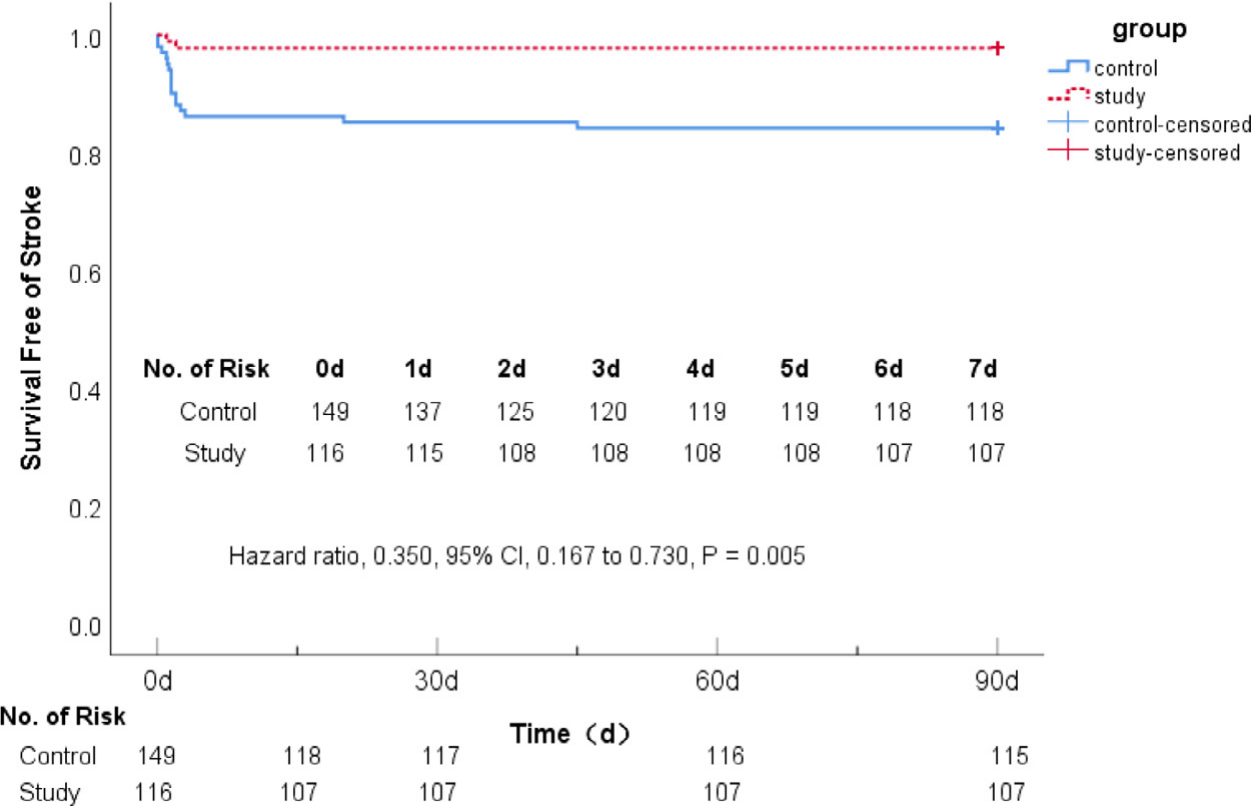
Comparison of recurrent ischemic stroke between the two groups within 90 days.

There were 34 (22.82%) patients in the control group and 9 (7.76%) patients in the study group with recurrent stroke events. The Cox proportional hazards model was used to evaluate the recurrence of ischemic stroke events between the two groups. The study group reduced the risk of recurrent cerebral infarction by 65% (Hazard Ratio [HR] 0.350; 95% CI 0.167–0.730, p = 0.005), showing that 7-day DAPT+intensive rosuvastatin could significantly reduce the risk of recurrent stroke for AIS patients within 90 days (Table 4 & Fig. 2).

**Table 4:** Comparison of recurrent ischemic stroke between two groups.

	Control group (n = 149)		Study group (n = 116)		HR and 95% CI	p-value
	Cases (*no*)	%	Cases (*no*)	%		
**Recurrent ischemic stroke**	34	22.82	9	7.76	0.350 (0.167‒0.730)	0.005
**Bleeding events**^a^	10	6.71	9	7.76	1.076 (0.424‒2.732)	0.878

a According to the GUSTO criteria, they were all minor gastrointestinal mucosal bleeding events.

### Comparison of bleeding events between two groups within 90 days

**Figure 3:**
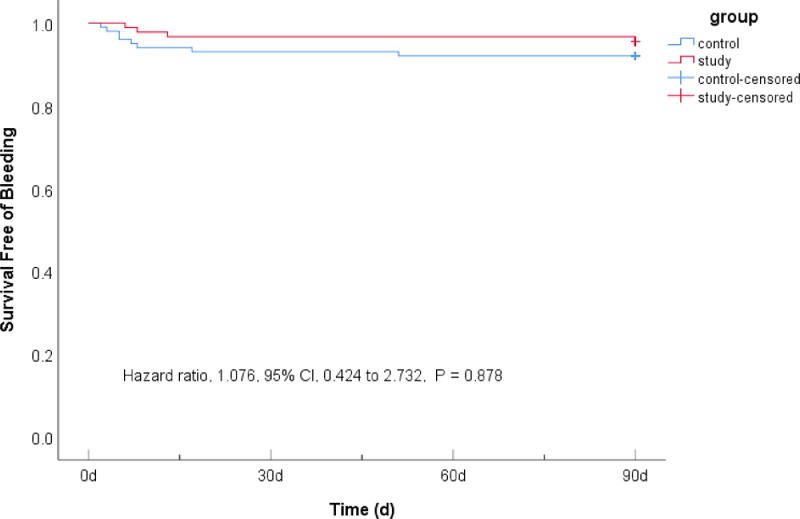
Probability of survival free of bleeding within 90 days.

There were 10 (6.71%) subjects in the control group and 9 (7.79%) in the study group with bleeding events, and there was no significant difference between the two groups (HR = 1.076, 95% CI 0.424–2.732, p = 0.878). No cases of cerebral hemorrhage were confirmed by head CT; mild gastrointestinal mucosal bleeding was confirmed by positive occult blood in stool and vomit,^12^ suggesting that 7-day DAPT+intensive rosuvastatin did not increase the risk of bleeding in patients with AIS (Table 4 & Fig. 3).

### SILI and SAM events

There was no occurrence of increased levels of ALT, AST, LDH, or CK by more than 3 times the upper limit of normal values within 90 days of follow-up. Comparing the levels of ALT, AST, LDH, and CK before and 2 weeks (14 ± 3 days) after treatment in the study group, no statistically significant difference was noted (p > 0.05), suggesting that intensive rosuvastatin rarely induced SILI or SAM (Table 5).

**Table 5:** Comparison of ALT, AST, LDH and CK before and 2 weeks after treatment.

		ALT (U/L)	AST (U/L)	LDH (U/L)	CK (U/L)
Before	25%	13.830	13.00	150.50	53.25
	Median	18.35	16.55	178.50	70.00
	75%	24.53	21.60	202.50	109.25
After	25%	12.65	14.10	151.00	46.50
	Median	17.20	17.70	171.50	68.00
	75%	25.58	21.85	202.25	101.50
p (2-tailed)		0.251	0.483	0.114	0.086

ALT, Alanine Transferase; AST, Aspartate Transferase; LDH, Lactate Dehydrogenase; CK, Creatine kinase.

## Discussion

Compared with the regimen of SAPT+rosuvastatin, short-term (7-day) DAPT+intensive rosuvastatin was an ideal regimen for mild-to-moderate AIS. Short-term DAPT+intensive rosuvastatin quickly and effectively relieved focal neurological deficits symptoms and significantly reduced the risk of recurrent ischemic stroke in patients with AIS within 90 days ‒ without increasing adverse events such as bleeding, SILI, or SAM. The results should be related to the strong inhibition of platelet aggregation and thrombosis reduction by DAPT, pleiotropy of intensive statins, and an appropriate DAPT course.

Activation and aggregation of platelets are major factors in thrombosis.^15^ Aspirin is a cyclooxygenase inhibitor, and clopidogrel is an adenosine diphosphate receptor antagonist. They can quickly and effectively inhibit platelet aggregation and platelet release through different ways, thereby reducing thrombosis and promoting thrombolysis. The DAPT with aspirin combined with clopidogrel is a drug recommended by the AIS guidelines,^10^ which can significantly reduce the risk of recurrent ischemic stroke in AIS patients.^16^^,^^17^ Non-cardiac ischemic stroke is attributed to Atherosclerotic Cardiovascular Disease (ASCVD),^18^ and the elevated levels of non-HDL-C and LDL-C in the circulation are the fundamental factors of atherosclerosis^18^, ^19^, ^20^ and the key to the occurrence of ASCVD events.^21^^,^^22^ Statins reduce endogenous cholesterol synthesis by inhibiting β-hydroxyβ-methylglutaryl-CoA reductase. They also increase non-HDL-C and LDL clearance by promoting the expression of low-density lipoprotein receptors concentrated in hepatocytes,^23^^,^^24^ which is an important means to reduce ASCVD events,^25^, ^26^, ^27^ and effectively reduce the risk of recurrent cerebral infarction.^28^^,^^29^ It is a routine drug for acute treatment^28^^,^^29^ and secondary prevention of ischemic stroke.^2^^,^^30^

Statins have other pleiotropic effects such as immunomodulation, improving endothelial function, and antioxidant and antithrombotic effects. By up-regulating Endothelial Nitric Oxide Synthase (eNOS), statins can increase nitric oxide concentration, dilate cerebral blood vessels, increase local cerebral blood flow, and relieve ischemia and hypoxia symptoms;^31^ Statins can increase blood supply to the ischemic zone by inhibiting thrombosis, promoting thrombolysis, and shrinking vascular plaques.^32^^,^^33^ Statins can reduce the infarct volume by promoting the formation of vascular structure and improving local blood supply;^34^ Statins exert their anti-inflammatory properties^35^, ^36^, ^37^ by inhibiting the release of inflammatory factors and reducing the stress response in the acute phase of ischemia.^38^ Statins can also improve endothelial function and inhibit apoptosis.^39^^,^^40^ All these effects are dose-dependent.^32^^,^^41^

Previous studies have shown that DAPT increased the occurrence of bleeding events,^7^, ^8^, ^9^ but this mainly occurred on the 8^th^–90^th^ day of DAPT;^9^ this study limited the therapy regimen to a 7-day course of DAPT to avoid bleeding events. Other studies showed that statins induced liver injury and elevated transaminase.^42^ However, rosuvastatin is a water-soluble statin, which can only enter the liver cells through specific channel proteins on the liver cell membrane to inhibit cholesterol synthesis. It is not easy to enter other tissues and cells, including striated muscle cells, and hence rosuvastatin rarely leads to side effects such as muscle weakness, soreness, and muscle enzyme elevation.^43^ This forms the basis of the therapy regimen of 7-day DAPT combined with intensive rosuvastatin.

The limitations of this study include its single-center and incomplete randomized controlled design, and its small sample size. The present results should therefore be interpreted with caution and validated further in future appropriately designed, large-scale studies.

In conclusion, short-term (7-d) DAPT combined with intensive rosuvastatin was a more accurate DAPT regimen for patients with mild-to-moderate AIS, which could rapidly and effectively relieve the clinical symptoms and significantly reduce the risk of recurrent ischemic stroke without increasing adverse events such as bleeding, SILI, or SAM.

## Funding

Supported by Beijing Municipal Commission of Science and Technology (Z181100001718066)

Trial Registration: China Clinical Trial Registry, ChiCTR1800017809 (2018-08-15).

## Author's contribution

Study conception or design: TZ and HTL; acquisition of the data: TD, JMC, XML, XHY, LSL; analysis and interpretation of the data: TD, WH, HTL; drafting and revising the article: TZ and HTL. All authors read and approved the final manuscript.

All authors approve to submit this manuscript.

## Conflicts of interest

The authors declare no conflicts of interest.
